# Reproductive Factors and Colorectal Cancer Risk: A Population-Based Case-Control Study

**DOI:** 10.1093/jncics/pkac042

**Published:** 2022-06-01

**Authors:** Efrat L Amitay, Tobias Niedermaier, Elizabeth Alwers, Jenny Chang-Claude, Michael Hoffmeister, Hermann Brenner

**Affiliations:** Division of Clinical Epidemiology and Aging Research, German Cancer Research Center (DKFZ), Heidelberg, Germany; Division of Clinical Epidemiology and Aging Research, German Cancer Research Center (DKFZ), Heidelberg, Germany; Division of Clinical Epidemiology and Aging Research, German Cancer Research Center (DKFZ), Heidelberg, Germany; Division of Cancer Epidemiology, German Cancer Research Center (DKFZ), Heidelberg, Germany; Genetic Tumor Epidemiology Group, University Medical Center Hamburg-Eppendorf, University Cancer Center Hamburg, Hamburg, Germany; Division of Clinical Epidemiology and Aging Research, German Cancer Research Center (DKFZ), Heidelberg, Germany; Division of Clinical Epidemiology and Aging Research, German Cancer Research Center (DKFZ), Heidelberg, Germany; Division of Preventive Oncology, German Cancer Research Center (DKFZ), Heidelberg, Germany; German Cancer Consortium (DKTK), German Cancer Research Center (DKFZ), Heidelberg, Germany

## Abstract

**Background:**

Hormone-replacement therapy (HRT) is associated with lower colorectal cancer (CRC) risk among postmenopausal women. However, little is known about the effects of lifetime exposure of women to varying levels of estrogen and progesterone through reproductive factors such as parity, use of oral contraceptives (OC), breastfeeding, and menstruation on CRC risk.

**Methods:**

We assessed associations between reproductive factors and CRC risk among 2650 female CRC patients aged 30+ years and 2175 matched controls in a population-based study in Germany, adjusting for potential confounders by multiple logistic regression.

**Results:**

Inverse associations with CRC risk were found for numbers of pregnancies (odds ratio [OR] per pregnancy = 0.91, 95% confidence interval [CI] = 0.86 to 0.97), breastfeeding for 12 months and longer (OR = 0.74, 95% CI = 0.61 to 0.90), and use of either OC or HRT (OR = 0.75, 95% CI = 0.64 to 0.87) or both (OR = 0.58, 95% CI = 0.48 to 0.70). Similar results were found for postmenopausal women only and when adjusting for number of pregnancies and for all reproductive factors analyzed together. Breastfeeding duration of 12 months and longer was associated with lower risk of cancer only in the proximal colon (OR = 0.58, 95% CI = 0.45 to 0.74).

**Conclusions:**

Several reproductive factors were associated with lower CRC risk in women, including number of pregnancies, breastfeeding duration, and use of OC and HRT. This suggests that women’s exposure to female reproductive hormones plays a key role in the difference in CRC risk between women and men and in site-specific CRC risk.

Colorectal cancer (CRC) is the second-most common cause of cancer death worldwide (1). The oncogenic effects of estrogens have been extensively investigated in breast cancer, but little is known about estrogen signaling in CRC, although CRC risk is lower in women compared with men (2-4).

Large population-based studies found that hormone-replacement therapy (HRT) was associated with lower CRC risk for postmenopausal women at all concentrations and durations of exposure (5,6). Although individual benefits of estrogen or progesterone are difficult to separate, the protective effects were also seen for estrogen treatment alone (7). Less is known about the association of lifetime exposure of women to varying levels of estrogen and progesterone through reproductive factors such as number of pregnancies, use of oral contraceptives (OC), breastfeeding, and menstruation with CRC risk.

A meta-analysis published in 2009 found that ever use of OC was associated with lower CRC risk (odds ratio [OR] = 0.81, 95% confidence interval [CI] = 0.72 to 0.92) (8). Results from the Women’s Health Initiative support the hypothesis that previous use of OC (ever vs never: hazard ratio [HR] = 0.74, 95% CI = 0.63 to 0.86) is associated with lower CRC risk. A similarly strong association was found for parity (2 children vs nulliparous: HR = 0.80, 95% CI = 0.64 to 0.99) (9). Results of the European Prospective Investigation into Cancer and Nutrition, a cohort study of over 500 000 participants from 10 European countries (10–12), support an inverse association between OC and CRC risk. However, no association was found with other reproductive factors such as age at menarche or menopause, parity, or breastfeeding (13). Similarly, in a cross-sectional Korean study, age at menarche, parity, use of OC or HRT, and menopause were not statistically significantly associated with risk of colorectal adenoma (14). Very few studies examined potential differences of association between reproductive factors and CRC by tumor location, and a meta-analysis published in 2013 did not find such differences (15). We investigated potential associations between reproductive factors and CRC risk among women in a large population-based case-control study from Germany.

## Methods

### Study Design and Population

The current analyses are based on data from the DACHS study (Darmkrebs: Chancen der Verhütung durch Screening / colorectal cancer: chances for prevention through screening), an ongoing population-based case-control study conducted in the Rhine-Neckar region of Germany originally designed to assess the potential of endoscopic screening for the prevention of CRC that is described elsewhere (16,17) (see the Supplementary Methods, available online, for details). The study was approved by the ethics committees of the University of Heidelberg and the state medical boards of Baden-Württemberg and Rhineland-Palatinate. All participants provided written informed consent. The current analyses included female participants recruited between 2003 and 2020. Comparison with data from population-based cancer registries suggests that approximately 50% of eligible patients (higher proportions among younger patients, lower proportions at oldest ages) were recruited. The participation rate of controls was 52% and followed the same age gradient as observed among cases.

### Data Collection

Patients were identified and informed about the study during first hospitalization for CRC surgery and interviewed by trained interviewers who collected sociodemographic, medical, and lifestyle information using standardized questionnaires. Patients who could not be recruited during hospital stay were contacted after discharge and interviewed at home. The median time between diagnosis and interview was 24 (interquartile range = 10-224) days. Controls were randomly selected from population registries; frequency matched to cases by age, sex, and county of residence; and contacted through the study center by mail and follow-up calls to schedule home interviews. Controls with a history of CRC were excluded; controls opting out of the interview were offered a self-administered short questionnaire.

### Assessment of Reproductive Factors

Lifetime reproductive factors were obtained during the interviews. We used 5 groups of reproductive exposure data in the current analyses: 1) pregnancies: number of pregnancies lasting 6+ months (>4 pregnancies were classified as “4+” pregnancies); 2) breastfeeding: ever breastfeeding, lifetime length of breastfeeding in months; 3) OC: ever use for 4+ months, age at start of using OC, years of use; 4) menstruation: age at menarche and menopause; and 5) HRT use after menopause. All information was collected retrospectively up to the time of the index date (cases: date of diagnosis; controls: date of interview).

### Statistical Analyses

We excluded CRC cases and control participants with missing information on pregnancies and OC use (n = 1049) and participants with Crohn’s disease or ulcerative colitis (n = 48; Supplementary Figure 1, available online). More controls than cases were excluded because of missing information on pregnancies and OC use because controls (unlike cases) could still participate in the study if they filled out a short questionnaire that did not cover those questions. In addition to analyses in the entire study population (N  = 4825), separate analyses were conducted among postmenopausal women (n = 4350) and women without previous colonoscopy (n = 2732). The rationale for the latter analysis was that previous colonoscopy with removal of CRC precursors might have eliminated much of the precolonoscopy hormonal exposure effects.

Logistic regression was used to estimate adjusted odds ratios and 95% confidence intervals for the association of reproductive factors with CRC risk. All models were adjusted for the following established CRC risk or preventive factors: age (cases: at diagnosis; controls: at interview), body mass index (BMI; continuous), education level (1-8, 9-10, >10 years), history of CRC in a first-degree relative (yes or no), previous large bowel endoscopy (ever or never), medical diagnosis of diabetes (yes or no), ever smoking regularly (never, stopped ≥2 years before joining the study, and smoking at the time of joining the study).

Each of the multivariable logistic regression models for overall CRC risk was adjusted for 1 of the reproductive exposures in the left column of Table 2 plus the following covariates. Model 1 included age (continuous; cases: at diagnosis; controls: at interview), BMI (continuous), family history of CRC (yes or no), past large bowel endoscopy (ever or never), ever smoking regularly (never, stopped ≥2 years before joining the study, smoking at the time of joining the study), education level (1-8, 9-10, >10 years), and medical diagnosis of diabetes (yes or no). Model 2 included age, BMI, history of CRC in a first-degree relative (yes or no), past large bowel endoscopy, smoking, education, and diabetes. Model 3 included age, BMI, family history of CRC, past large bowel endoscopy, smoking, education, diabetes, and age at first and last pregnancy (above or below median). In the first set of analyses, covariate adjusted associations with CRC risk were assessed in separate models for each reproductive factor individually. In a second (model 2) and third set of analyses (model 3), the various reproductive factors were jointly included. Model 3 differs slightly from model 2 in variable definitions and selection (HRT and OC use were considered in a combined variable; age at first and last pregnancy were additionally included). Logistic regression was used to estimate adjusted odds ratios and 95% confidence intervals for the association of reproductive factors with CRC risk.

Multinomial logistic regression was used to examine the association of reproductive factors on CRC risk by tumor location: proximal vs distal colon and colon vs rectum. Participants with missing values in the variables included in the final model (all <1% missing except BMI, with 1.5% missing) were excluded from the analyses. Statistical tests were 2-sided with an α level of .05. All analyses were conducted using R (18).

## Results

### Study Participants

Our analyses included 2650 women diagnosed with CRC in 2003-2020 and 2175 controls without history of CRC recruited during the same years (Table 1). Cases tended to be less educated, have higher BMI, and were more often smokers. Among cases, there was a greater proportion of participants with a family history of CRC, 29% of the cases and 61% of the controls had previous endoscopy, and a larger proportion of cases were diagnosed with diabetes.

**Table 1.** Characteristics of study population

Characteristics	Cases, No. (%) (n = 2650)	Controls, No. (%) (n = 2175)	*P* ^a^
Mean age (SD), y	69.5 (11.6)	68.6 (11.5)	.01
Age, y			.048
<50	141 (5.3)	117 (5.4)	
50 to <65	991 (37.4)	733 (33.7)	
65 to <75	699 (26.4)	630 (29.0)	
≥75	819 (30.9)	695 (32.0)	
BMI, kg/m^2^			<.001
<25	1065 (41.1)	1073 (49.7)	
25-30	984 (37.9)	761 (35.2)	
>30	544 (21.0)	325 (15.1)	
Education, y			<.001
1-8	1703 (64.5)	1152 (53.1)	
9-10	578 (21.9)	587 (27.1)	
>10	361 (13.7)	431 (19.9)	
Smoking			.002
Never	1665 (63.2)	1437 (66.1)	
Past	598 (22.7)	505 (23.2)	
Current	370 (14.1)	232 (10.7)	
First-degree family history of CRC			<.001
No	2232 (84.3)	1910 (87.9)	
Yes	415 (15.7)	263 (12.1)	
Previous large bowel endoscopy			<.001
No	1878 (70.9)	854 (39.3)	
Yes	771 (29.1)	1321 (60.7)	
Diabetes			<.001
No	2165 (82.4)	1922 (88.7)	
Yes	462 (17.6)	246 (11.3)	
Ever use OC			<.001
No	1349 (51.0)	872 (40.1)	
Yes	1294 (49.0)	1301 (59.9)	
Ever pregnant			.62
No	302 (11.9)	265 (12.4)	
Yes	2242 (88.1)	1878 (87.6)	
Ever breastfeeding			.06
No	728 (31.2)	547 (28.6)	
Yes	1605 (68.8)	1368 (71.4)	
Ever HRT use			<.001
No	1908 (72.5)	1300 (59.9)	
Yes	725 (27.5)	870 (40.1)	
HRT and OC use			<.001
Never	1039 (39.6)	560 (25.8)	
One	1167 (44.4)	1047 (48.3)	
Both	421 (16.0)	561 (25.9)	
Median age at menarche, y			.77
<14	1140 (44.4)	941 (44.0)	
≥14	1425 (55.6)	1198 (56.0)	
Age at menarche, y			.27
<12	219 (8.5)	150 (7.0)	
12 to <14	921 (35.9)	791 (37.0)	
14	704 (27.4)	586 (27.4)	
≥15	721 (28.1)	612 (28.6)	
Menopause			.07
No	242 (9.1)	233 (10.7)	
Yes	2408 (90.9)	1942 (89.3)	
Recruitment year			.001
2003-2008	1002 (37.8)	770 (35.4)	
2009-2014	865 (32.6)	654 (30.1)	
2015-2020	783 (29.5)	751 (34.5)	

a Fisher’s exact test, except for mean difference in age (*t* test); numbers in table may not amount to N  = 4825 due to missing values. BMI = body mass index; CRC = colorectal cancer; HRT = hormone replacement therapy; OC = oral contraceptives.

### Reproductive Factors and CRC Risk

**Figure 1.**
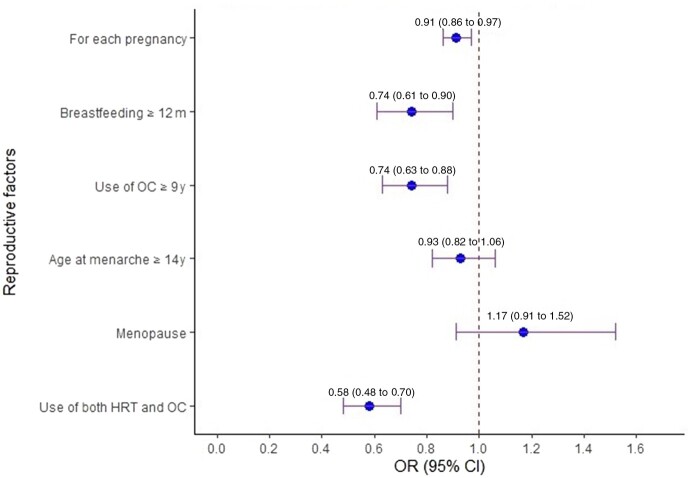
Reproductive factors and colorectal cancer (CRC) risk: entire study population (N = 4825). **Error bars** represent the 95% confidence intervals. CI = confidence interval; HRT = hormone replacement therapy; OC = oral contraceptives; OR = odds ratio.

In the entire study population (Table 2; Figure 1), pregnancies were inversely related to CRC risk (OR = 0.91 per pregnancy, 95% CI = 0.86 to 0.97). Cumulative breastfeeding duration of 12 months or longer was associated with lower CRC risk (OR = 0.74, 95% CI = 0.61 to 0.90) compared with never breastfeeding, whereas age at menarche was not associated with CRC risk. OC use was associated with lower CRC risk, with a stronger association for use of 9 years and longer (OR = 0.74, 95% CI = 0.63 to 0.88). Use of OC or HRT (1 of them) was associated with 25% lower CRC risk (OR = 0.75, 95% CI = 0.64 to 0.87); use of both was associated with a 42% lower CRC risk (OR = 0.58, 95% CI = 0.48 to 0.70).

**Table 2.** Reproductive factors and CRC risk: multivariable logistic regression models

Reproductive factors	No. of cases	No. of controls	Model 1^a^	Model 2^b^	Model 3^c^
			OR (95% CI)	OR (95% CI)	OR (95% CI)
No. of pregnancies lasting 6+ mo, for each pregnancy			0.91 (0.86 to 0.97)	0.94 (0.87 to 1.00)	0.93 (0.87 to 0.99)
Breastfeeding duration, mo					
No	707	541	Reference	Reference	Reference
<12	1180	1013	0.97 (0.83 to 1.13)	0.98 (0.83 to 1.15)	0.98 (0.84 to 1.15)
≥12	423	417	0.74 (0.61 to 0.90)	0.75 (0.61 to 0.93)	0.76 (0.62 to 0.94)
Use of OC					
No	1280	855	Reference	Reference	—
<9 y^d^	581	601	0.77 (0.64 to 0.92)	0.77 (0.64 to 0.93)	—
≥9 y	671	675	0.74 (0.63 to 0.88)	0.79 (0.66 to 0.94)	—
Age at menarche, y					
<14	1097	928	Reference	Reference	Reference
≥14	1382	1182	0.93 (0.82 to 1.06)	0.94 (0.82 to 1.09)	0.92 (0.80 to 1.05)
Menopause					
No	236	233	Reference	Reference	Reference
Yes	2310	1911	1.17 (0.91 to 1.52)	1.29 (0.95 to 1.73)	1.27 (0.95 to 1.70)
Ever HRT use					
No	1908	1300	—	Reference	—
Yes	725	870	—	0.72 (0.62 to 0.84)	—
Ever HRT and OC use					
None	984	552	Reference	—	Reference
One	1137	1029	0.75 (0.64 to 0.87)	—	0.71 (0.60 to 0.84)
Both	410	556	0.58 (0.48 to 0.70)	—	0.55 (0.45 to 0.68)

a Model 1 includes: one of the reproductive exposures in the column and also the covariates: age (continuous), BMI (continuous), family history of CRC (yes or no), past large bowel endoscopy (ever or never), smoking (current, former, or never), education (3 levels), and diabetes (yes or no). BMI = body mass index; CI = confidence interval; CRC = colorectal cancer; HRT = hormone replacement therapy; OC = oral contraceptives; OR = odds ratio.

b Model 2 includes all the reproductive factors in the column and also the covariates: age, BMI, family history of CRC, past large bowel endoscopy, smoking, education, and diabetes.

c Model 3 includes all the reproductive factors in the column and also the covariates: age, BMI, family history of CRC, past large bowel endoscopy, smoking, education, diabetes, and age at first and last pregnancy (above or below median). Pregnancies were counted continuously for up to 4 pregnancies; >4 pregnancies was counted as 4 pregnancies to avoid inconsistent estimates caused by outliers.

d Dichotomized at the median duration of OC use.

### CRC Risk Among Postmenopausal Women

In postmenopausal women (n = 4350), additionally adjusted for HRT, results were similar (model 1, Supplementary Table 1, available online). Each pregnancy lasting 6 months and longer was associated with reduced CRC risk by 8% (OR = 0.92, 95% CI = 0.87 to 0.98). Lifetime breastfeeding duration of 12 months and longer (OR = 0.75, 95% CI = 0.61 to 0.92), OC use (OR = 0.75, 95% CI = 0.63 to 0.90), and age at menopause of 50 years old or older (OR = 0.83, 95% CI = 0.73 to 0.96) were associated with lower CRC risk whereas age at menarche was not.

### CRC Risk Among Postmenopausal Women: Adjusted for Number of Pregnancies

Because the number of pregnancies was highly associated with CRC risk and was associated with OC use and breastfeeding, we repeated the analysis while further adjusting it among postmenopausal women for the number of pregnancies in addition to HRT use (model 2; supplementary Table 1, available online). The results remained very similar: breastfeeding (≥12 months) was associated with an approximately 20% lower CRC risk (OR = 0.80, 95% CI = 0.64 to 0.99) as was OC use (OR = 0.79, 95% CI = 0.66 to 0.95) and age of menopause of 50 years old or older (OR = 0.82, 95% CI = 0.71 to 0.94). The results were virtually identical when adjusting for all factors simultanously (model 3; Supplementary Table 1, available online).

### CRC Risk Among Women Without Previous Large Bowel Endoscopy

We repeated the analyses among women with no previous large bowel endoscopy (n = 2732) because previous colonoscopy with removal of colorectal neoplasms might have eliminated much of the precolonoscopy hormonal exposure effects (Supplementary Table 2, available online). Results were similar and remained statistically significant for the association between lower CRC and OC use (≥9 years: OR = 0.77, 95% CI = 0.60 to 0.99), HRT use (OR = 0.70, 95% CI = 0.56 to 0.87) and the use of both OC and HRT (OR = 0.53, 95% CI = 0.40 to 0.71). Menopausal status was associated with increased CRC risk (OR = 1.52, 95% CI = 1.07 to 2.15). The association of pregnancies with lower CRC risk was similar to the association observed in the entire study population but was no longer statistically significant, probably due the smaller sample size (OR = 0.92, 95% CI = 0.84 to 1.00). Smaller comparison groups may have also affected the results regarding the association of breastfeeding and CRC risk.

### CRC Risk by Tumor Location

**Figure 2.**
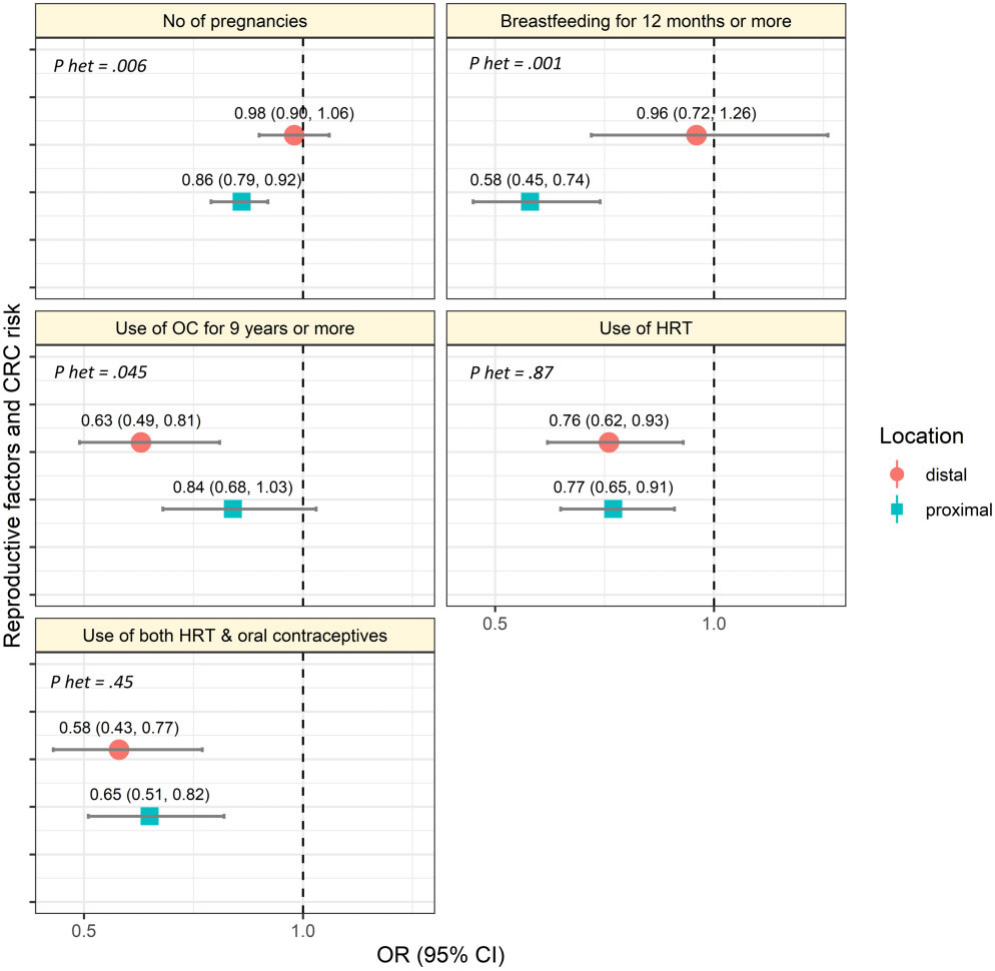
Reproductive factors and colorectal cancer (CRC) risk by tumor location in the colon. **Error bars** represent the 95% confidence intervals. CI = confidence interval; HRT = hormone replacement therapy; OC = oral contraceptives; OR = odds ratio; *P*-het = *P*_heterogeneity_ from case-case analysis.

We compared associations between proximal and distal colon cancers (Figure 2; Table 3). Breastfeeding for 12 months and longer was associated with lower cancer risk only in the proximal colon (OR = 0.58, 95% CI = 0.45 to 0.74 for proximal and OR = 0.96, 95% CI = 0.72 to 1.26 in the distal colon, *P*_heterogeneity_ = .001). Conversely, OC use for 9 years and longer was associated with lower cancer risk only in the distal colon (OR = 0.63, 95% CI = 0.49 to 0.81 for distal and OR = 0.84, 95% CI = 0.68 to 1.03 for proximal, *P*_heterogeneity_ = .045). HRT was associated with lower risk irrespective of tumor location (OR = 0.76, 95% CI = 0.62 to 0.93 for proximal and 0.77, 95% CI = 0.65 to 0.91 in the distal colon). No statistically significant differences were found in the association with other reproductive factors between tumor locations. In further analyses, no statistically significant differences were found in the association of reproductive factors and risk of cancer in the colon and the rectum (Supplementary Table 3, available online).

**Table 3.** Reproductive factors and CRC risk by tumor location in the colon^a^

Reproductive factors	Controls, No. (%)	Proximal colon		Distal colon		*P*_heterogeneity_ ^b^
		No. (%)	OR (95% CI)	No. (%)	OR (95% CI)	
No. of pregnancies lasting 6+ mo for each	1907	913	0.86 (0.79 to 0.92)	586	0.98 (0.90 to 1.06)	.006
No. of pregnancies lasting 6+ mo						
0	263 (12.5)	116 (12)	Reference	84 (13)	Reference	
1	481 (22.9)	280 (28)	1.23 (0.94 to 1.63)	156 (24)	1.00 (0.72 to 1.37)	.28
2	840 (40.0)	373 (38)	0.94 (0.73 to 1.22)	241 (37)	0.86 (0.64 to 1.16)	.73
3	359 (17.1)	153 (15)	0.83 (0.61 to 1.13)	99 (15)	0.76 (0.54 to 1.08)	.78
>3	157 (7.5)	70 (7)	0.65 (0.45 to 0.95)	67 (10)	0.92 (0.61 to 1.37)	.12
Breastfeeding duration, mo						
No	540 (28.8)	316 (35)	Reference	168 (29)	Reference	
<12	916 (48.9)	436 (49)	0.84 (0.70 to 1.02)	279 (49)	1.07 (0.85 to 1.34)	.07
≥12	417 (22.3)	140 (16)	0.58 (0.45 to 0.74)	127 (22)	0.96 (0.72 to 1.26)	.001
Use of OC (median)						
No	852 (41.6)	541 (55)	Reference	336 (53)	Reference	
<9 y	501 (24.4)	187 (19)	0.83 (0.67 to 1.05)	141 (22)	0.80 (0.72 to 1.26)	.83
≥9 y	697 (34.0)	260 (26)	0.84 (0.68 to 1.03)	152 (24)	0.63 (0.49 to 0.81)	.045
Age at menarche, y (median)						
<14	924 (43.9)	431 (43.5)	Reference	300 (46)	Reference	
≥14	1181 (56.1)	560 (56.5)	0.91 (0.77 to 1.07)	348 (54)	0.89 (0.74 to 1.08)	.76
Menopause						
No	232 (10.8)	54 (5.3)	Reference	62 (9)	Reference	
Yes	1907 (89.2)	964 (94.7)	1.32 (0.91 to 1.93)	600 (91)	1.22 (0.83 to 1.79)	.79
Use of HRT						
No	1300 (59.9)	747 (70.3)	Reference	495 (73)	Reference	
Yes	870 (40.1)	316 (29.7)	0.77 (0.65 to 0.91)	187 (27)	0.76 (0.62 to 0.93)	.87
Ever HRT and OC use						
None	552 (25.8)	416 (40.9)	Reference	254 (38)	Reference	
One	1029 (48.2)	424 (41.7)	0.76 (0.62 to 0.92)	302 (46)	0.77 (0.62 to 0.97)	.93
Both	556 (26.0)	176 (17.3)	0.65 (0.51 to 0.82)	105 (16)	0.58 (0.43 to 0.77)	.45

a Multinomial logistic regression model includes: age (continuous), BMI (continuous), family history of CRC (yes or no), past large bowel endoscopy (ever or never), smoking (current, former, or never), education (3 levels), diabetes (yes or no), and 1 of the reproductive exposures in the table. CI = confidence interval; CRC = colorectal cancer; HRT = hormone replacement therapy; OC = oral contraceptives; OR = odds ratio.

b *P*_heterogeneity_: case-case analysis.

## Discussion

In this large German population-based case-control study, we found statistically significant associations between female reproductive factors and lower risk of CRC. Longer duration of breastfeeding, pregnancies, OC and HRT use, and older age at menopause were associated with a lower risk of CRC. Age at menarche was not associated with CRC risk. Major differences were found in the association of reproductive factors with risk of cancer in the proximal and distal colon.

Several previous studies have investigated the association between female reproductive factors and CRC risk. In line with our study, age at menarche was not associated with CRC risk in a meta-analysis from 2013 (19), which was confirmed more recently (20). Researchers in another study using genetic risk scores based on single-nucleotide polymorphisms associated with age at menarche and menopause as surrogates for endogenous estrogen exposure found no statistically significant associations of genetic risk scores for age at menarche (OR per year = 0.98, 95% CI = 0.95 to 1.02) and age at menopause (OR = 0.98, 95% CI = 0.94 to 1.01) with CRC risk (21). The authors of one study (22) found increased risk for distal colon cancer with later age at menarche, earlier age at menopause, and more full-term pregnancies. Researchers of a later study (23), however, did not find age at menarche to be associated with risk of proximal or distal colon, or rectum cancer, in line with our findings. Overall, there is no convincing evidence for an association between age at menarche and risk of CRC.

To disentangle the effects of breastfeeding from those of pregnancy (because breastfeeding can only apply to parous women), we conducted sensitivity analyses restricted to women with at least 1 pregnancy lasting 6+ months. The results were virtually identical to our main analyses, with odds ratios being mostly only less than 1% lower or higher (maximum: 1.5%). Thus, breastfeeding appeared to be associated with CRC risk independently of pregnancy.

The authors of a meta-analysis from 2013 also found no association between parity and CRC risk (15), with no differences by colonic site. However, in the UK-based Million Women Study, parity was associated with lower CRC risk (risk ratio [RR] = 0.91, 95% CI = 0.87 to 0.95) but no trend was found for number of births (24). Considering these results, we assume that the association between parity and CRC risk is at best very small. Parity increases estrogen levels approximately 10-fold (15) but may also increase insulin secretion to compensate for a decreased glucose tolerance (15). While higher estrogen levels suggest CRC protective effects, increased risk is indicated for higher insulin levels (25). In sum, those opposing effects might largely offset each other, potentially limiting the effect of parity on CRC risk. In line with this hypothesis, our study suggested no association between parity (ever vs nulliparous) and CRC risk (OR = 0.96, 95% CI = 0.80 to 1.16).

Few studies have looked into the association between breastfeeding and CRC risk. The authors of an Egyptian study from 2010 found that breastfeeding for 19 months and longer per live birth was associated with lower CRC risk (OR = 0.2, 95% CI = 0.1 to 0.4) (26). Other studies found no association (13,23,27). Only 1 previous study (23) provided stratified analysis by tumor location and found a lower risk with breastfeeding only for proximal colon cancer (HR = 0.65, 95% CI = 0.43 to 0.99) compared with null associations for distal colon and rectum cancer, which is in line with our results. Location-specific differences in the proximal colon might also point to a role of molecular tumor subtypes, such as microsatellite instability (MSI-high) and *BRAF* mutation (28).

A meta-analysis from 2001 showed that ever use of OC was associated with lower CRC risk (RR = 0.81, 95% CI = 0.69 to 0.94), similar to our study, though no association was found with duration of use (29). Potential differences by duration of use and tumor location suggested by our study need confirmation from other studies and further analyses by CRC molecular subtypes. No statistically significant association was found with OC use by Nichols et al. (30) with other reproductive factors, including age at menarche, parity, and age at menopause. Reduced risk of CRC associated with HRT was previously demonstrated in DACHS (6) and other studies (31,32), although null associations were also found (33). The authors of a previous meta-analysis found differences in the association of HRT use and CRC risk by MSI status (34). Risk reduction was similar with respect to MSI status in a larger study from 2020 (6), but risk reduction with the predominantly proximal CRC subtypes (MSI-high, *BRAF* mutation, CIMP-high, sessile serrated pathway CRC) was only observed in the age group older than 71 years.

It may seem conflicting that both pregnancies and OC use were associated with lower risk of CRC. However, OCs prevent pregnancies by elevating progesterone levels, just as an actual pregnancy would, that is, by “imitating” the presence of a pregnancy.

Researchers of 2 previous studies (22,24) simultaneously investigated reproductive factors. Burón Pust et al. (24) found lower CRC risk among parous women (RR = 0.91, 95% CI = 0.87 to 0.95), like our study, but no association with number of births. OC use was not associated with CRC risk, irrespective of duration of use. Yoo et al. (22) suggested from analyses among 372 CRC cases and 21 061 controls that history of full-term pregnancy was strongly inversely associated with CRC risk, whereas older age at first pregnancy or full-term pregnancy and at menopause were associated with higher risk. For other reproductive factors, no statistically significant associations were found. Also, Nichols et al. (30) found no associations between CRC risk and age at menarche or menopause but did find a lower risk for women with age at first birth above the median (23 years) and a lower rectal cancer risk for 5 and more births compared with nulliparous women.

The mechanisms behind the association between CRC risk in women and reproductive factors are unclear. Effects are difficult to discern from each other because exposures such as parity, breastfeeding, and OC use are connected. The association of HRT and lower CRC risk also has been found in many previous studies and underpins the role of estrogen in CRC development that results from estrogen receptors (ERs, mainly ERβ) in the colonic epithelium (35). Exposure to estrogen can also arise from OC use, younger age at menarche, older age at menopause and pregnancy, with the latter increasing both progesterone and estrogen levels.

Estrogen affects the cells in the colon mainly through ERβ, which has antiproliferative effects (21). The results of a review (36) showed that despite expression of ERβ in normal colonocytes, none of the colon cancer cell lines expressed a sufficient amount of ERβ, suggesting that loss of expression of ERβ is part of tumorigenesis. Furthermore, it was hypothesized that increased local concentration of estrogens in the colon reduces production of carcinogenic secondary bile acid, limits DNA damage and MSI, and inhibits cell proliferation of colonic tumors (27).

Inverse associations between HRT and CRC risk are consistent with previous studies, most importantly the Women’s Health Initiative (WHI) trial, which showed a hazard ratio of 0.63 (95% CI = 0.43 to 0.92) with vs without combined estrogen and progestin intake (5). Nevertheless, given clinically relevant increases in risk for other outcomes (HRs for coronary heart disease: 1.29, breast cancer: 1.26, stroke: 1.41), HRT is not a suitable strategy for CRC prevention among women.

The strengths of this study are its large size, the population-based design, comprehensive assessment of the reproductive factors, and adjustment for other lifestyle, medical, and family history exposures. To our knowledge, this is the first study to investigate such a wide selection of reproductive factors in association with site-specific (and not only overall) CRC risk.

Limitations include the case-control design and self-reported data (though during standardized interviews). Many exposure variables and covariates referred to time periods decades ago, making imperfect or imprecise recall conceivable. An example is years of OC use, for which a higher than expected number of women reported 5, 10, 15, etc years of use (“end-digit preference”). Differential recall of lifetime exposures between cases and controls is unlikely, however, suggesting that exposure–disease associations might have been underestimated due to nondifferential misclassification. Selection bias could have occurred if, for example, more health conscious controls with favorable risk factor distribution were more likely to participate in the study than cases with a similar distribution. However, we did not focus on “classical” risk factors, such as smoking, overweight or obesity, etc, but on hormonal factors that are not linked to health consciousness in the general perception, making relevant selection bias unlikely. Nonparticipation in this study, which did not have an upper age limit, was most strongly related to old age among both cases and controls, making major selection bias unlikely. Given the high mean age at diagnosis of female CRC patients (75 years in Germany), not excluding a large proportion of CRC patients simply due to older age can also be considered of major importance with respect to external validity.

In conclusion, several reproductive factors were statistically significantly associated with CRC risk in women, including number of pregnancies, breastfeeding duration, OC use, and HRT use. Those findings could help to develop risk-adapted screening strategies based on established determinants of CRC that could additionally consider factors assessed in this study, for example, by prolonging CRC screening intervals among women with protective factors such as having had 3 or more pregnancies. Our findings are largely in line with the hypothesis that higher lifetime estrogen levels (be it from OCs, HRT, or pregnancies) are associated with lower CRC risk and may point to a part played by women’s reproductive hormones in the difference in CRC risk between women and men. Ideally, lifetime exposure to estrogens and also potentially confounding factors such as insulin and insulin-like growth factor levels would be repeatedly assessed in a future large prospective study, with a particular focus on estrogen levels during lifetime. This could enable a more comprehensive investigation of the suggested mechanisms behind the observed associations.

## Funding

This work was supported by the German Research Council (grant number BR 1704/6–1, BR 1704/6–3, BR 1704/6–4, BR 1704/6–61, CH 117/1–1, HO 5117/2–1, HE 5998/2–1, KL 2354/3–1, RO 2270/8–1; and BR 1704/17–1), the German Federal Ministry of Education and Research (grant number 01KH0404, 01ER0814, 01ER0815, 01ER1505A; and 01ER1505B), and the Interdisciplinary Research Program of the National Center for Tumor Diseases (NCT), Germany.

## Notes

**Role of the funder:** The funder had no role in the design of the study; the collection, analysis, and interpretation of the data; writing of the manuscript or decision to submit it for publication.

**Disclosures:** All authors declare that there are no conflicts of interest to disclose.

**Author contributions:** Conceptualization: EAm, JCC, HB, MH; Data curation: EAm, JCC, HBr, MH; Methodology: EAm, TN, EAl, JCC, MH, HB; Formal analysis: EAm, TN; drafting of the manuscript: EAm, TN; Funding acquisition: HB, MH; Supervision: JCC, HBr, MH; Writing—review and editing: all authors; approval of the final version of the manuscript: all authors.

**Acknowledgements:** The authors thank Ute Handte-Daub for her excellent technical assistance. The authors thank the study participants and the interviewers who collected the data. The authors also thank the following hospitals and cooperating institutions that recruited patients for this study: Chirurgische Universitätsklinik Heidelberg, Klinik am Gesundbrunnen Heilbronn, St. Vincentiuskrankenhaus Speyer, St. Josefskrankenhaus Heidelberg, Chirurgische Universitätsklinik Mannheim, Diakonissenkrankenhaus Speyer, Krankenhaus Salem Heidelberg, Kreiskrankenhaus Schwetzingen, St. Marienkrankenhaus Ludwigshafen, Klinikum Ludwigshafen, Stadtklinik Frankenthal, Diakoniekrankenhaus Mannheim, Kreiskrankenhaus Sinsheim, Klinikum am Plattenwald Bad Friedrichshall, Kreiskrankenhaus Weinheim, Kreiskrankenhaus Eberbach, Kreiskrankenhaus Buchen, Kreiskrankenhaus Mosbach, Enddarmzentrum Mannheim, Kreiskrankenhaus Brackenheim, and Cancer Registry of Rhineland-Palatinate, Mainz.

## Data Availability

The data underlying the research results described in the manuscript can be made available upon reasonable request.

## Supplementary Material

pkac042_Supplementary_Data
