# Supplementary material for: Reproductive Factors and Colorectal Cancer Risk: A Population-Based Case-Control Study
Source: JNCI Cancer Spectr. 2022 Jun 1;6(4):pkac042. doi: 10.1093/jncics/pkac042 (PMC9251386; doi:10.1093/jncics/pkac042)

Supplementary Materials

Reproductive factors and colorectal cancer risk: Population-based case-control study

Authors: Efrat L Amitay*, Tobias Niedermaier*, Elizabeth Alwers, Jenny Chang-Claude, Michael Hoffmeister, Hermann Brenner

*contributed equally

Supplementary methods

Study design and population

The current analyses are based on data from the DACHS study, an ongoing population-based case-control study conducted in the Rhine-Neckar region of Germany, originally designed to assess the potential of endoscopic screening for the prevention of CRC, and described elsewhere [13, 14]. In short, since 2003, patients with a first histologically confirmed diagnosis of CRC and randomly selected population-based control participants with no history of CRC are recruited by all (>20) hospitals providing CRC surgery for a population of ~2 million people in the study region located in south-western Germany. Controls and CRC patients are frequency-matched by 5-year age group, sex and county of residence.

Supplementary Table 1: Reproductive factors and CRC risk among post-menopausal women only

Reproductive Factors		N Cases	N Controls	Model 1[a] OR (95%CI)	Model 2[b] OR (95%CI)	Model 3[c] OR (95%CI)
Number of pregnancies lasting 6+ months	For each pregnancy			0.92 (0.87, 0.98)	—	—
Number of pregnancies lasting 6+ months	0	263	220	Reference	—	Reference
	1	587	444	1.09 (0.86, 1.38)	—	2.34 (0.79, 6.92)
	2	835	727	0.94 (0.75, 1.17)	—	2.09 (0.71, 6.15)
	3	365	327	0.83 (0.64, 1.07)	—	1.93 (0.65, 5.74)
	>3	198	153	0.75 (0.55, 1.01)	—	1.79 (0.59, 5.43)
Breastfeeding duration in months	No	651	509	Reference	Reference	Reference
	<12	1081	928	1.00 (0.85, 1.18)	1.01 (0.86, 1.19)	1.03 (0.86; 1.23)
	≥12	363	334	0.75 (0.61, 0.92)	0.80 (0.64, 0.99)	0.82 (0.65; 1.04)
Use of oral contraceptives in years	No	1237	823	Reference	Reference	Reference
	<9	424	419	0.78 (0.65, 0.94)	0.80 (0.66, 0.98)	0.80 (0.65, 0.98)
	≥9	545	581	0.75 (0.63, 0.90)	0.79 (0.66, 0.95)	0.78 (0.64, 0.96)
Age at menarche in years	<14	951	789	Reference	Reference	Reference
	≥14	1286	1086	0.92 (0.80, 1.06)	0.91 (0.79, 1.05)	0.93 (0.80, 1.09)
Age in years at menopause	<50	1064	836	Reference	Reference	Reference
	≥50	1138	1002	0.83 (0.73, 0.96)	0.82 (0.71, 0.94)	0.80 (0.69, 0.94)

[a]Logistic regression model 1 includes: age (continuous), BMI (continuous), family history of CRC (yes/no), past large bowel endoscopy (ever/never), smoking (current/former/never), education (3 levels), HRT use (yes/no), diabetes (yes/no) and one of the reproductive exposures in the table.

[b] Logistic regression model 2 includes: age, BMI, family history of CRC, past large bowel endoscopy, smoking, education, HRT use, diabetes, number of pregnancies lasting 6+ months and one of the reproductive exposures in the table.

[c] Logistic regression model 3 includes all the variables included in model 2 and all the reproductive factors listed (simultaneous adjustment for all variables) with pregnancies of 6+ months included as categorical variable.

Supplementary Table 2: Reproductive factors and CRC risk among women without previous large bowel endoscopy

Reproductive Factors		N Cases	N Controls	Model 1[a] OR (95%CI)	Model 2[b] OR (95%CI)
Number of pregnancies lasting 6+ months	For each pregnancy			0.92 (0.84, 1.00)	0.92 (0.85, 1.01)
Breastfeeding duration in months	No	205	495	Reference	Reference
	<12 months	322	737	1.00 (0.80, 1.26)	1.01 (0.81, 1.26)
	≥12 months	205	336	0.83 (0.63, 1.10)	0.84 (0.64, 1.11)
Ever oral contraceptive use	No	339	921	Reference	—
	<9 years	203	349	0.77 (0.59, 1.00)	—
	≥9 years	273	465	0.77 (0.60, 0.99)	—
Age at menarche in years	<14 years	392	769	Reference	Reference
	≥14 years	437	984	1.09 (0.89, 1.32)	1.04 (0.86, 1.26)
Menopause	No	166	204	Reference	Reference
	Yes	674	1601	1.52 (1.07, 2.16)	1.52 (1.07, 2.15)
HRT use	No	589	1383	Reference	—
	Yes	251	422	0.70 (0.56, 0.87)	—
Ever HRT and oral contraceptives use	None	257	748	—	Reference
	One	414	806	—	0.75 (0.59, 0.94)
	Both	169	248	—	0.53 (0.40, 0.71)

[a]Logistic regression model 1 includes all the reproductive factors in the column and also the covariates: age (continuous), BMI (continuous), family history of CRC (yes/no), smoking (current/former/never), education (3 levels) and diabetes (yes/no).

[b]Model 2 includes all the reproductive factors in the column and also the covariates: age, BMI, family history of CRC, smoking, education and diabetes.

Supplementary Table 3: reproductive factors and CRC risk by tumor location: colon versus rectum[a]

Reproductive Factors		Controls N (%)	Colon		Rectum		P heterogeneity[b]
			N (%)	OR (95%CI)	N (%)	OR (95%CI)	
Number of pregnancies lasting 6+ months	For each pregnancy	1907	913	0.9 (0.85, 0.96)	586	0.94 (0.87, 1.02)	0.233
Number of pregnancies lasting 6+ months	0	263 (13)	202 (12)	Reference	99 (12)	Reference	
	1	481 (23)	437 (27)	1.12 (0.88, 1.42)	214 (26)	1.25 (0.92, 1.68)	0.395
	2	840 (40)	619 (38)	0.90 (0.72, 1.13)	308 (37)	0.97 (0.73, 1.28)	0.447
	3	359 (17)	252 (15)	0.77 (0.60, 1.00)	136 (16)	0.95 (0.69, 1.32)	0.122
	>3	157 (7)	137 (8)	0.73 (0.54, 1.01)	70 (8)	0.89 (0.60, 1.32)	0.325
Breastfeeding duration in months	No	540 (29)	485 (33)	Reference	218 (30)	Reference	
	<12 months	916 (49)	719 (49)	0.92 (0.78, 1.09)	364 (50)	1.08 (0.88, 1.34)	0.149
	≥12 months	417 (22)	268 (18)	0.70 (0.56, 0.86)	153 (21)	0.82 (0.64, 1.07)	0.231
Use of oral contraceptive use	No	852 (42)	881 (54)	Reference	394 (49)	Reference	
	<9 years	501 (24)	330 (20)	0.80 (0.66, 0.97)	165 (20)	0.70 (0.55, 0.90)	0.330
	≥9 years	697 (34)	413 (25)	0.73 (0.61, 0.87)	251 (31)	0.78 (0.62, 0.98)	0.491
Age at menarche in years	<14 years	924 (44)	736 (45)	Reference	358 (44)	Reference	
	≥14 years	1181 (56)	911 (55)	0.88 (0.77, 1.02)	464 (56)	1.06 (0.89, 1.26)	0.069
Menopause	No	232 (11)	118 (7)	Reference	118 (14)	Reference	
	Yes	1907 (89)	1570 (93)	1.25 (0.92, 1.68)	729 (86)	1.23 (0.89, 1.71)	0.850
Ever HRT and oral contraceptives use	None	552 (26)	671 (40)	Reference	313 (37)	Reference	
	One	1029 (48)	732 (43)	0.76 (0.64, 0.89)	405 (48)	0.73 (0.59, 0.89)	0.621
	Both	556 (26)	282 (17)	0.61 (0.50, 0.75)	128 (15)	0.52 (0.40, 0.68)	0.271

[a] Multinomial logistic regression model includes: age (continuous), BMI (continuous), family history of CRC (yes/no), past large bowel endoscopy (ever/never), smoking (current/former/never), education (3 levels), diabetes (yes/no) and one of the reproductive exposures in the table.

[b]P-value for heterogeneity: case-case analysis

Supplementary Figure 1: Study inclusion

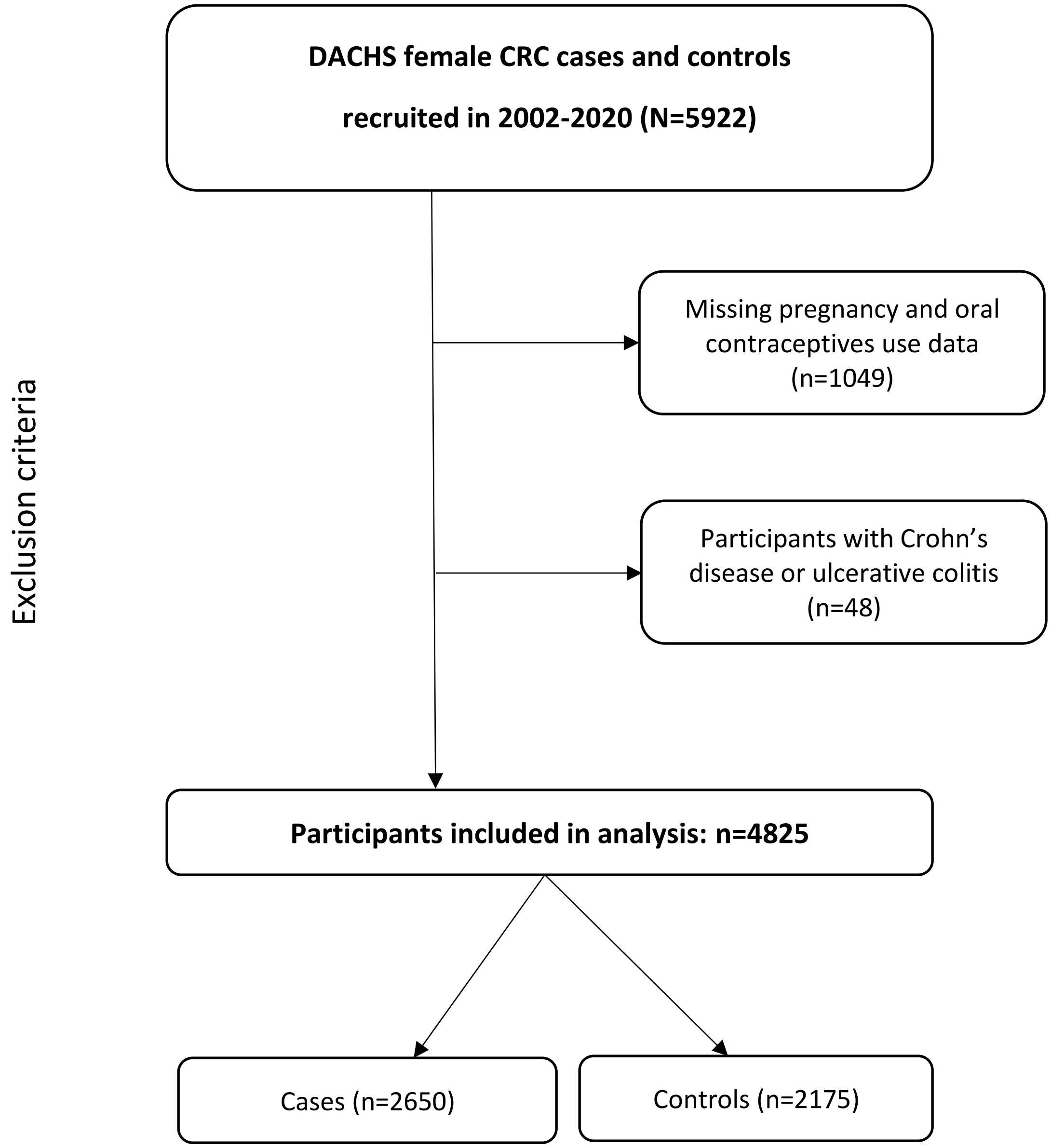

Supplement: pkac042_Supplementary_Data [file pkac042_supplementary_data.pdf]
